# Application of 3D tooth model for monitoring of implant space and inter-root distance without radiographs: a proof of concept study

**DOI:** 10.1186/s40729-020-00253-3

**Published:** 2020-09-30

**Authors:** Kyungmin Lee, Gyu-Hyoung Lee

**Affiliations:** Department of Orthodontics, School of Dentistry, Chonnam National University, 33 Yongbong-ro, Buk-gu, Gwangju, 61186 South Korea

**Keywords:** 3D tooth model, CBCT, Intraoral scan, Root position, Radiation

## Abstract

**Background:**

Radiographs are integral in evaluating implant space and inter-root distance. The purpose of this report is to introduce a method for evaluating the 3D root position with minimal radiation using a 3D tooth model composed of an intraoral-scanned crown and a cone-beam computed tomography (CBCT)-scanned root.

**Materials and methods:**

Intraoral scan and CBCT scan of the patient were obtained before treatment. In the CBCT image, tooth segmentation was performed by isolating individual teeth from the maxillary and mandibular alveolar bone using software program. The 3D tooth model was fabricated by combining segmented individual teeth with the intraoral scan.

**Results:**

A post-treatment intraoral scan was integrated into the tooth model, and the resulting position of the root could be predicted without additional radiographs. It is possible to monitor the root position after a pretreatment CBCT scan using a 3D tooth model without additional radiographs.

**Conclusion:**

The application of the 3D tooth model benefits the patient by reducing repeated radiation exposure while providing the clinician with a precise treatment evaluation to monitor tooth movement.

## Background

Successful treatment depends not only on the initial diagnosis but also on the accurate assessment of treatment progress. Clinicians need to perform cone-beam computed tomography (CBCT) scanning to monitor and evaluate tooth movement, especially root movement. CBCT can depict the true root position and 3-dimensional (3D) angulation and permits accurate evaluation of tooth movement. Although the radiation dose used in dental imaging is far less than that in imaging in other spheres of medicine, multiple CBCT scans are not recommended due to the public’s and patients’ increasing concerns about radiation hazard [1].

The failure to sequentially assess the root position leads to unexpected treatment time delay and undesired results. The purpose of this report was to suggest a 3D method to monitor tooth position without additional CBCT scans. The method uses *3D tooth models* composed of an intraoral-scanned crown and a CBCT-scanned root. The patient’s intraoral and CBCT scans were obtained before treatment. In the CBCT image, the tooth was isolated from the alveolar bone using a software program. By combining isolated individual teeth and intraoral scans, tooth models composed of intraoral-scanned crowns and CBCT-scanned roots were fabricated. When there was a need to evaluate the root position during treatment, the patient’s dentition was scanned using an intraoral scanner. Additional intraoral scans at any stage of treatment were integrated into the tooth models, and the resulting position of the root was predicted without a CBCT scan. Based on the tooth models, clinicians could evaluate 3D tooth movement during treatment with only intraoral scans and without additional CBCT scans. The purpose of this report is to introduce a method to evaluate 3D root position with minimal radiation using a 3D tooth model composed of an intraoral-scanned crown and a CBCT-scanned root.

## Materials and methods

**Fig. 1:**
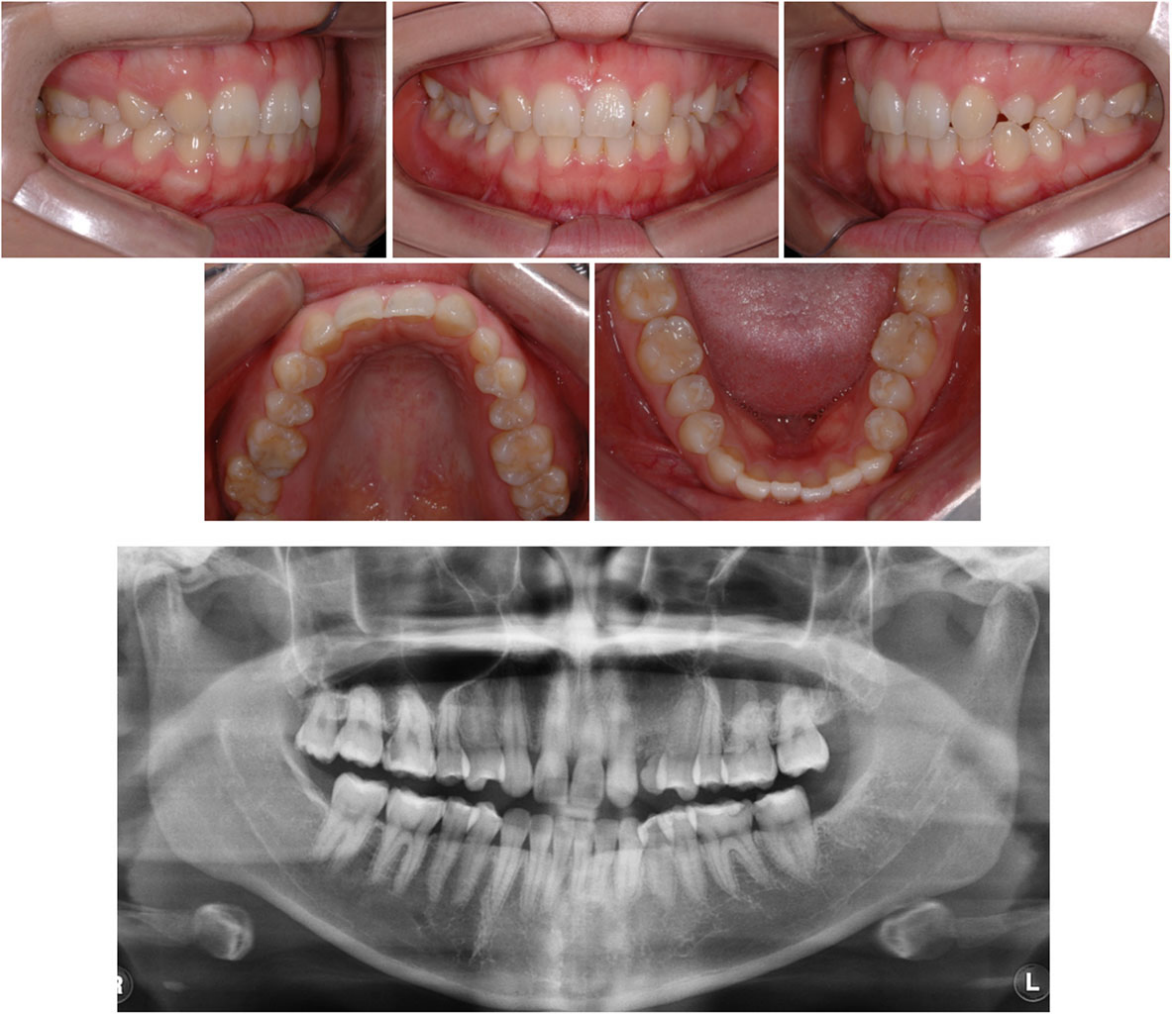
Pretreatment intraoral photographs and panoramic radiograph

**Fig. 2:**
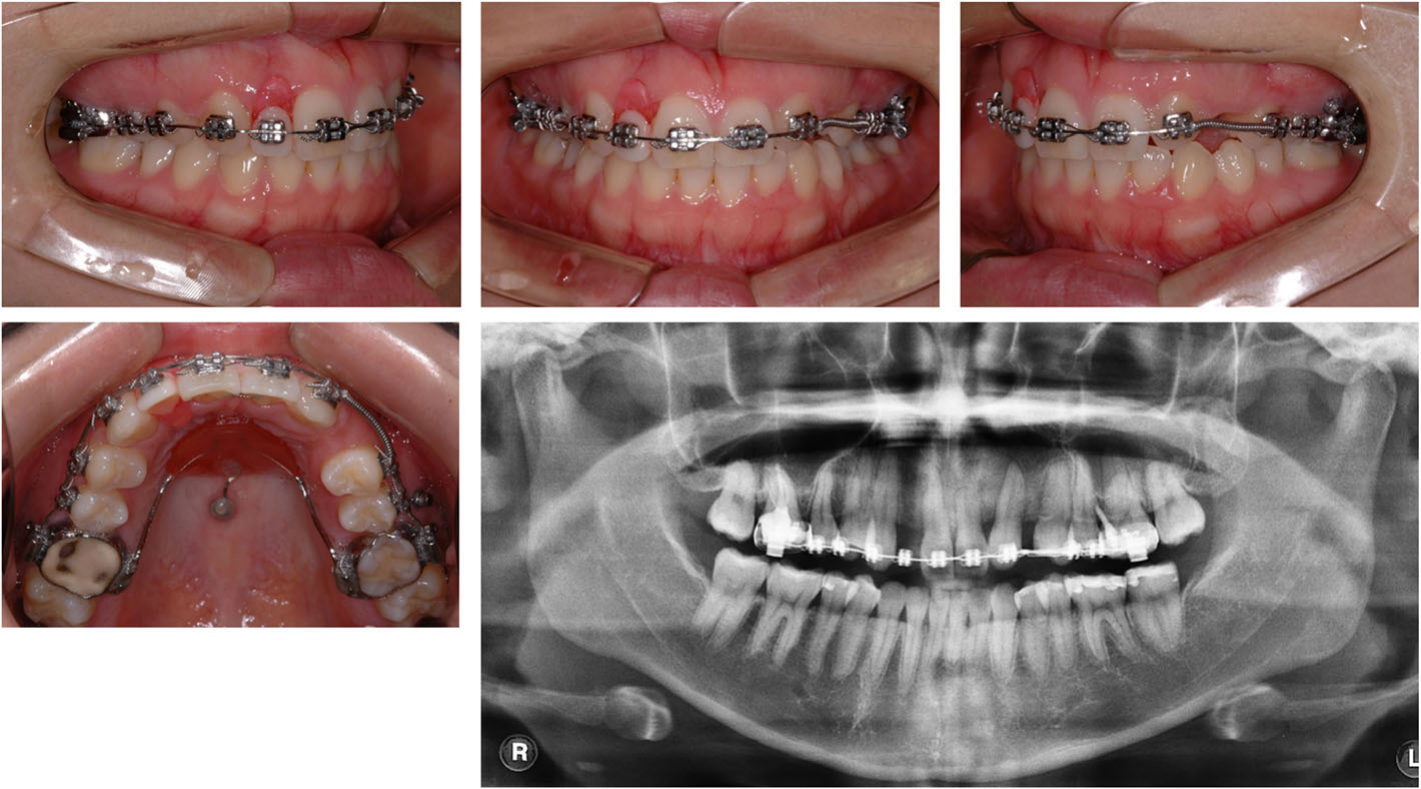
Intraoral photographs and panoramic radiographs after 9 months of space opening treatment

A 23-year-old female patient visited the Department of Orthodontics at Chonnam National University with the chief complaint of midline discrepancy. Intraoral photographs showed a midline discrepancy and retained left deciduous canine (Fig. 1). On pretreatment panoramic radiographs, we observed congenitally bilateral missing lateral maxillary incisors and the left deciduous canine was retained in the canine area (Fig. 1). CBCT (Alphard Vega; Asahi Roentgen, Kyoto, Japan; 80 kV and 5 mA; voxel size, 0.39 mm × 0.39 mm × 0.39 mm; and field of view, 200 mm × 179 mm) was obtained in the Department of Prosthodontics to evaluate the available space for the implants. After treatment planning with a prosthodontist, space opening for prosthetic restoration of the maxillary lateral incisors was decided. Concerning the root positions of the maxillary central incisors and canines, the space opening was planned to the right lateral incisor area and left canine area. On the right side, the canine would be retracted to gain space for the right lateral incisor implant, and for the left side, lateralization of the canine and space gaining was planned. After 9 months of orthodontic treatment, the patient was referred to the Department of Prosthodontics for implant space evaluation. The prosthodontist evaluated the implant space using panoramic radiographs and suggested gaining more space in the left implant area (Fig. 2). The ideal distance from the base of the contact point to the alveolar bone crest between a tooth and an implant is 3 to 5 mm [2, 3]. However, panoramic radiographs may be inaccurate in showing the true root positions. Previous studies have shown that panoramic radiographs contain inherent image distortions, and the assessment of root parallelism using panoramic radiography should be performed with caution, especially in premolar extraction sites [4, 5]. Thus, an additional CBCT scan was needed to accurately determine the space for the lateral incisor and canine, but the patient’s radiation exposure was of concern.

### Pretreatment fabrication of the 3D tooth model

Intraoral and CBCT scans of the pretreatment record were used to fabricate the tooth models. Intraoral scans of the maxillary and mandibular arches were obtained using a TRIOS scanner (3Shape, Copenhagen, Denmark), and scan data were converted into stereolithography (STL) format using the OrthoAnalyzer^TM^ software (3Shape). The CBCT scan data were imported into InVivo5 software (version 5.1, Anatomage, San Jose, CA) for tooth segmentation. In the “MD (medical design) studio” module, the teeth were isolated from the alveolar bone, segmented individually using the “sculpt” function, and converted into the STL file format.

**Fig. 3:**
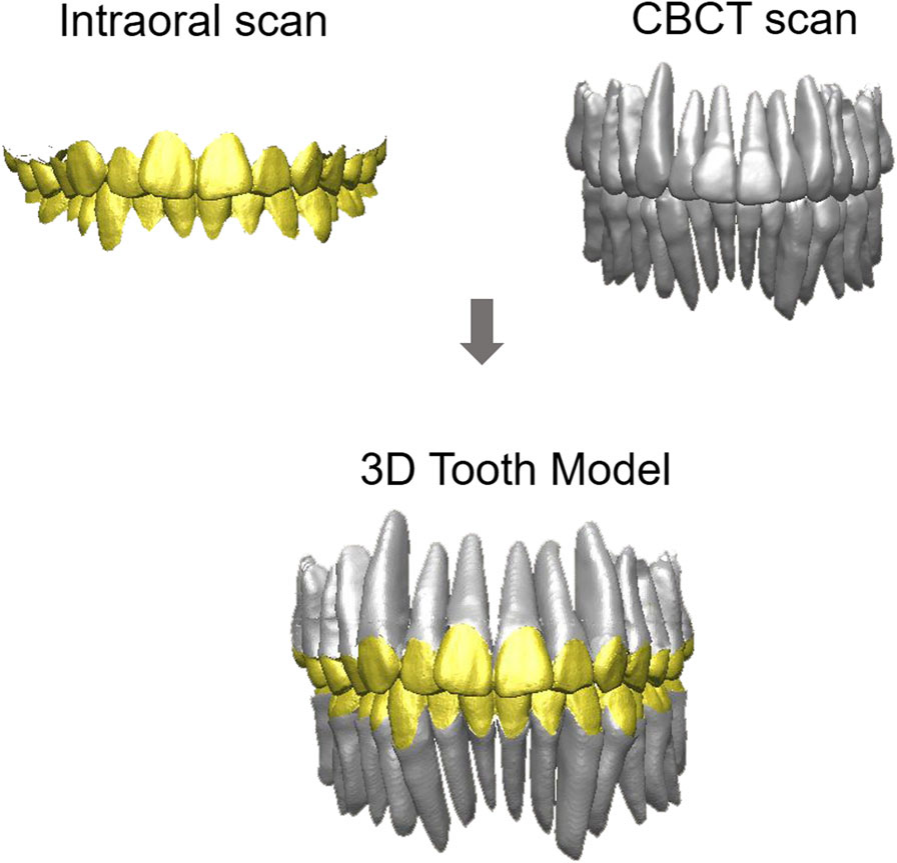
The concept of this method using a 3D tooth model

Segmented teeth, including the root from the CBCT scan and intraoral scan data, were imported into the Rapidform^TM^ 2006 (3D Systems, RockHill, SC). The CBCT scan and intraoral scan data were integrated using the registration function of the software. Initial registration was performed by selecting more than three corresponding points of each image, resulting in a rough alignment. Then, a “fine” automatic best-fit registration was used to finalize the matches. In order to fabricate the tooth model composed of an intraoral-scanned crown and CBCT-scanned root, the area of the CBCT-scanned crown was removed from the integrated image, and finally, the intraoral-scanned crown and CBCT-scanned root were merged using the program (Fig. 3).

## Results

**Fig. 4:**
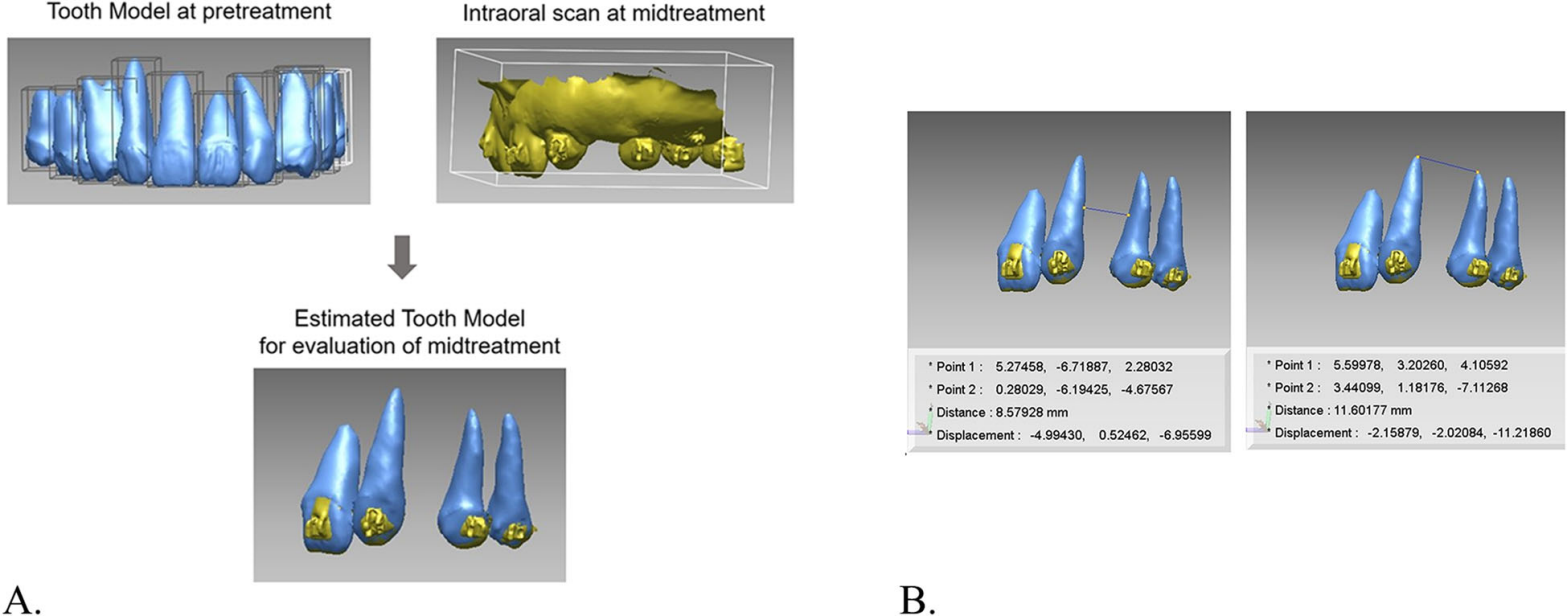
Generation of a tooth model with expected root position using the tooth model at pretreatment and intraoral scan at mid-treatment. **a** Intraoral scan at mid-treatment was registered onto the individual tooth model at pretreatment. **b** On the software program, the measured inter-root distance was 8.6 mm at the mid-root and 11.6 mm at the apex

**Fig. 5:**
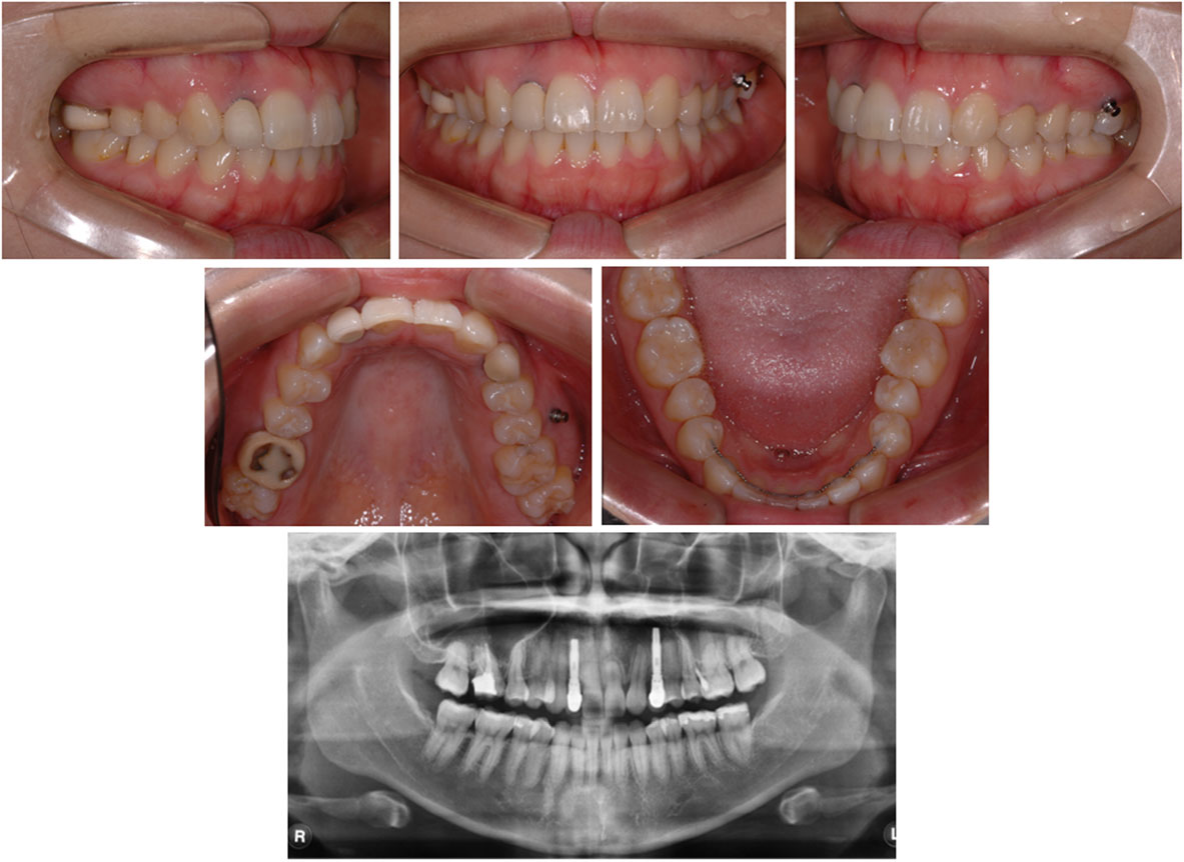
Post-treatment intraoral photographs and panoramic radiograph after implant restoration

**Fig. 6:**
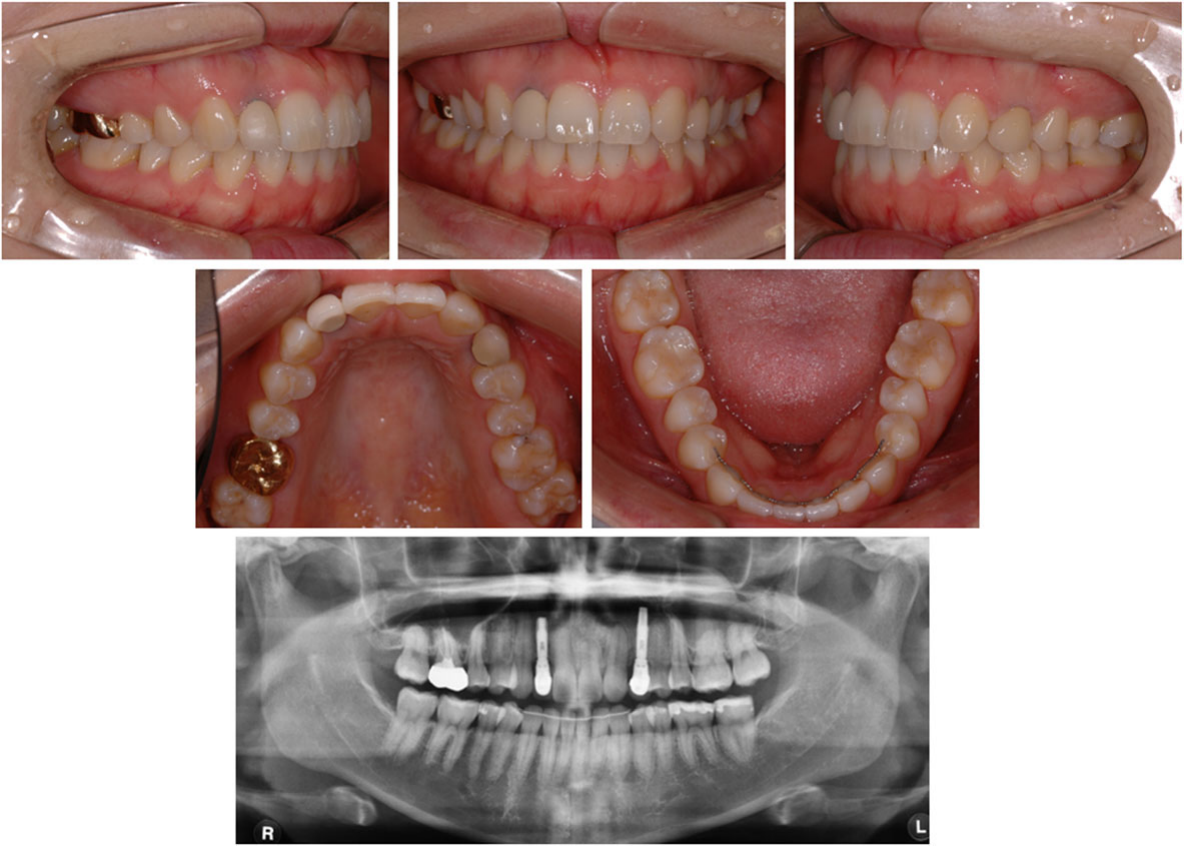
Two-year post-treatment intraoral photographs

After consulting a prosthodontist, the patient’s maxillary left quadrant area was scanned using an intraoral scanner. Mid-treatment intraoral scan data (9 months of orthodontic treatment) were registered onto the tooth models at pretreatment. The resulting position of the root was estimated without additional CBCT scans. The inter-root distance was evaluated (Fig. 4a). The space between the fixture and the adjacent roots seemed too close on the panorama; however, there was enough space for a fixture. The space was 8.6 mm at the mid-root level and 11.6 mm at the apex (Fig. 4b). This indicated that more space regaining was not needed. The patient started prosthetic restoration treatment without additional orthodontic treatment (Fig. 5). Without additional CBCT scans, 3D root positions were evaluated, and unnecessary additional treatment resulting in an undesired delay in the treatment period was prevented. After 2 years of post-treatment, the intraoral photographs and panoramic radiograph showed stable implants with good esthetic and occlusal results (Fig. 6).

## Discussion

Integrating intraoral scans and CBCT images by replacing the dental part of a CT image with an intact dental image allows a precise evaluation of 3D tooth movement in clinical applications where 3D information of the root is needed, including tooth arrangement, tooth movement monitoring, and orthodontic treatment simulation. Artifacts on CBCT images may be due to beam hardening and the enamel [6], which can appear because of differences in the radiation attenuation coefficient of the natural dentition without metal restoration. These artifacts may influence the accuracy of integration when CBCT and intraoral scans are superimposed. As CBCT images do not accurately represent occlusion, especially the occlusal surface in dental images [7], replacing the dental part of a CT image with an intact dental image is essential for the accurate fabrication of 3D tooth models [8, 9].

In this report, our method first fabricated tooth models after pretreatment by merging CBCT- and an intraoral-scanned crowns at pretreatment; then, this was used for the sequential evaluation of tooth movement. When integrating intraoral scan and CBCT images, accurate registration of the two images is essential for the high quality of the integrated image. The fabrication of the tooth model includes two steps: segmentation of the tooth in the CBCT image and merging the CBCT tooth root with an intraoral-scanned crown. The tooth segmentation process, which is separating an individual tooth from the alveolar bone on the CBCT images, is a fundamental step in the fabrication of tooth models. This process requires considerable time and labor, but with the advent of software programs, the segmentation of teeth on CBCT images is becoming simple and less laboring. Recent software programs use artificial intelligence technology for tooth segmentation. Many third-party software programs have been used for imaging processing. The need for an advanced program for accurate tooth segmentation/isolation is required in future research.

In order to apply this method to clinical situations, initial scan data (CBCT scan and intraoral scan) at pretreatment must be present. Thus, the sequential evaluation of tooth root position, such as implant restoration treatment and orthodontic treatment, is needed and is the best indication for this method.

Individual tooth models can be used to guide clinicians to anticipate root movement. The basic concept of this method is to estimate the tooth root position with only intraoral scanning without additional CBCT scanning. For example, during orthodontic treatment with premolar extraction, clinicians often experience tilting of the root around the extracted space. In this case, the evaluation of the root position is possible without additional CBCT scanning and with only intraoral scanning. In the case of adolescent patients who are awaiting implant installation with the implant space maintained, when the implant is to be installed a few years later, the inter-root space needs to be evaluated. At that time, the use of this method using only intraoral scanning enables the evaluation of inter-root space without additional CBCT scanning.

The innovative point of this method is to fabricate the 3D virtual tooth model by combining intraoral-scanned crown and CBCT-scanned root, that is called the composed tooth model. To the best of our knowledge, there are no software programs to make this composed tooth model at once. Since several programs and steps are used, fabrication of the tooth model is complicated at present, but it is hoped that a method that can be produced more easily will be developed due to the development of technology in the future. This technique provides a way to look at 3D tooth movement and may provide a better predictability. The application of the 3D tooth model benefits the patient by reducing radiation exposure while providing the clinician with a precise treatment evaluation to monitor tooth movement.

## Conclusion

The application of the 3D tooth model benefits the patient by reducing radiation exposure while providing the clinician with a precise treatment evaluation to monitor tooth movement.

## Data Availability

The datasets included in the current study are available from the corresponding author upon valid request.
